# Correction for Kincaid et al., “Noncanonical MicroRNA (miRNA) Biogenesis Gives Rise to Retroviral Mimics of Lymphoproliferative and Immunosuppressive Host miRNAs”

**DOI:** 10.1128/mBio.01413-20

**Published:** 2020-09-01

**Authors:** Rodney P. Kincaid, Yating Chen, Jennifer E. Cox, Axel Rethwilm, Christopher S. Sullivan

**Affiliations:** a The University of Texas at Austin, Molecular Biosciences, Austin, Texas, USA; b Institut für Virologie und Immunbiologie, Universität Würzburg, Würzburg, Germany

## AUTHOR CORRECTION

Volume 5, no. 2, e00074-14, 2014, https://doi.org/10.1128/mBio.00074-14. R. P. Kincaid, Y. Chen, and C. S. Sullivan would like to note the following. In follow-up confirmatory studies, we have attempted to repeat the work presented in Fig. 5B. We are unable to locate the original cell line reagents used in this work. We therefore attempted to regenerate similar cell lines to repeat this work. We attempted to generate stable cell lines in the GM19240 B-cell background, but we have repeatedly failed to generate these stable cell lines. These particular LCL cells appear to be refractory to efficient lentiviral transduction, and therefore, we conclude that the data forming the foundation of the original interferon-suppression-of-growth assay (Fig. 5B) are not reliable. Thus, although we have confirmed that miR-S6S7 can modulate effects of type I interferon on an ISRE reporter in cells, we conclude that we have no reliable evidence regarding the activity of miR-S6S7 on cell growth.

However, major conclusions from the original manuscript remain valid. These include the following: (i) simian foamy viruses (SFVs) encode miRNAs, (ii) SFV miRNAs arise via a noncanonical mode of biogenesis, and (iii) two SFV miRNAs share sequence identity and functionality in regulating target transcripts in common with the lymphoproliferative and immunosuppressive host miRNAs miR-155 and miR-132.

We apologize for and deeply regret the errors in this publication. C. S. Sullivan, the principal investigator of the Sullivan lab and senior author, accepts full responsibility for allowing these errors to be included in the original paper.

